# Microbial Musings – January 2021

**DOI:** 10.1099/mic.0.001033

**Published:** 2021-01-29

**Authors:** Gavin H. Thomas

**Affiliations:** ^1^​ Department of Biology, University of York, York YO10 5YW, UK

As we start the new year, the threat of infectious disease is still very much in the forefront of the public’s mind, to a level not seen in at least a generation or more. Microbiologists play important roles in both the research required to understand the infectious agent, a virus in this case, and to communicate this to both policy-makers and the public. Perhaps a long-term consequence of the outbreak could be that global issues that were ignored as ‘other people’s problems’ now get the attention they deserve, and to that end in this month we publish a series of Personal Views relating to other areas where microbiology can make a significant impact. These tie in directly to the United Nations Sustainable Development Goals (UN-SDGs) and link to an exercise undertaken by the Microbiology Society through a series of workshops to bring together experts in the three identified areas, namely antimicrobial resistance (AMR), soil health and the circular economy. Certainly, the latter two of these are closely interconnected, as the circular economy requires the use of sustainable, i.e. plant-based, feedstocks for manufacturing and the productivity of this process is intricately linked to soil health. All of them require fundamental understanding of microbial function, which is the overriding focus of articles published in *Microbiology*, and the application of this knowledge to develop, for example, a new antimicrobial, assemble an effective bioinoculant or improve the production of chemicals using microbial fermentation. I particularly like the argument made in the article by Geertje van Keulen about soil health, in that while the public are very conscious of air and water quality, which are indeed protected by various government directives, soil is not considered in the same way, yet is critically important to agricultural productivity and is significantly impacted by microbial function.

Another major bit of news for the journal for 2021 is the appointment of 21 new editors serving to support the journal during their term by encouraging and handling submissions and managing our new series of collections that we are launching to link to the 75th Anniversary of the journal in 2022 (Table 1). I am delighted by this significant new set of appointments for the journal, many of whom are specifically supporting our new topic areas announced last month. We will be revamping the website to help you, as potential authors, to identify an editor more easily with the right expertise to handle your next submission.

**Table 1.** The 21 new editors of *Microbiology* for 2021

Name	Organization	Twitter	Country
Jessica Blair	University of Birmingham	@JessicaMABlair	UK
David Clarke	University College Cork	@cleverflick	Ireland
Gautam Dey	European Molecular Biology Laboratory (EMBL)	@Dey_Gautam	Germany
Katherine Duncan	University of Strathclyde	@kate_duncan	UK
Jenna Gallie	Max Planck Institute for Evolutionary Biology	@gallie_jenna	Germany
Jamie Hall	University of Liverpool	@jpjhall	UK
Nicola Holden	Scotland’s Rural College	@NicolaJHolden	UK
Matt Hutchings	John Innes Centre	@MattHutchings10	UK
Nathalie Juge	Quadram Institute	@JugeLab	UK
Kimberley Kline	Nanyang Technological University	@KimInSingapore	Singapore
Ayush Kumar	University of Manitoba	@ayushkumarLab	Canada
Jacob Malone	John Innes Centre		UK
Despoina Mavridou	University of Texas at Austin	@bondSSbond	USA
Daniel Neill	University of Liverpool		UK
Pedro Oliveira	Genoscope		France
Kai Papenfort	Friedrich Schiller University of Jena	@papenfortlab	Germany
Nichollas Scott	University of Melbourne	@Nick_E_Scott	Australia
Nick Tucker	University of Strathclyde	@Tucker303	UK
Meera Unnikrishnan	The University of Warwick	@MeeraUnnikri	UK
Marjan Van der Woude	University of York		UK
Stineke Van Houte	University of Exeter	@StinekevanHoute	UK

We start this month with an interesting review and update on the progress of a ‘living antibiotic’. Anybody who has been paying attention over the last decade will have heard the inspirational Liz Sockett (@Bdello_Lab_Nott) from the University of Nottingham, UK, talking about her favourite bacterium *Bdellovibrio bacteriovorus *, a parasitic bacterium that attaches, invades and then grows in the bacterial periplasm (Bdella from the Greek for leech). I first remember reading about this mysterious microbe in John Postgate’s ‘Microbes and Man’, as being akin to some intermediate between a virus and a bacterium due to its small size and parasitic lifestyle. In fact, Postgate clearly was not entirely sure what it was, stating that ‘Bdellovibrios are probably true bacteria’ in his early editions, waiting until the fourth edition of 2000 to remove the ‘probably’! The first formal description of the lysis of Gram-negative bacteria by *B. bacteriovorus* was published in this journal in 1965 from Moshe Shilo and Barbara Bruff [1], working in Roger Stanier’s lab at UC Berkeley, which had followed shortly after its first formal description in 1963 [2, 3]. Liz and others have been making great strides in deciphering the lifecycle of ‘Bdello’, recognized by her election as a Fellow of the Royal Society in 2019. Her colleagues Robert Atterbury (@AtterburyLab) and Jess Tyson, both also at the University of Nottingham, have been leading research aimed more directly at testing the potential of Bdello to be clinically useful in killing pathogens, and the review in this issue assesses the current landscape and progress on this journey [4]. On paper there are many positives for Bdello, such as the lack of any known resistance mechanisms by the prey cells, a relatively broad spectrum compared to bacteriophage and fairly rapid killing in the laboratory, but do they actually work in the human body? The authors first present various studies suggesting that they are safe to use. Tests with animal cells in culture suggest that the bacteria only induce a weak inflammatory response, possibly due to its unusual lipopolysaccharide structure and sheathed flagella, and administration to several animal models appears to have no detrimental effect. Evidence is then presented to assess whether they can actually kill bacterial pathogens *in vivo,* and an early study from the authors demonstrated protection of chickens from *Salmonella* infections [5]. The review discusses other supportive studies and some that are more ambiguous, which are shedding light on the host range in which the bacteria can work ‘in the field’. As the authors discuss in the rest of the review, there is still much to do to take this unique treatment through the many stages required to get a new medicine to market, as well as persuading the public that taking one bacterium to kill another is a good idea, although in the current climate there is a strong argument to keep investigating and developing all possible routes to new anti-infectives.

This month we also have the Microbe Profile of the hyperthermophilic bacterium *Aquifex aeolicus* [6]. Thermophily has been something that has always fascinated me, namely how bugs avoid getting boiled, and while many of these hyperthermophiles are Archaea, such as *Sulfolobus solfataricus* that my colleague at the University of York, Daniella Barilla (@daniela_barilla), is studying, there are plenty of bacteria too. The most famous of these is *Thermus aquaticus* discovered by Thomas D. Brock and colleagues in the 1960s at Yellowstone National Park [7], which of course was the source of *Taq* DNA polymerase familiar to all molecular biologists. *Aquifex aeolicus* was a bacterium I first came across in 1998 in the early days of genomics, at a time when a bacterial genome was still novel enough to command a centre page spread on the pages of *Nature* or *Science* [8]. Its genome was novel as it was small, at around 1.5 Mb, and yet encoded what was needed to live a challenging high-temperature lifestyle at 95 °C. The genome contained many genes with evolutionary origins in Archaea and was the first example of apparently extensive long-distance horizontal gene exchange [9]. In this article from Marianne Guiral and Marie-Thérèse Guidici-Orticoni from the CNRS Laboratory of Bioenergetics and Protein Engineering in Marseille, France, the authors outline very clearly the plethora of interesting metabolic features that this bacterium can achieve [6]. As well as being a thermophile, it is also a chemolithotroph, using both molecular hydrogen and inorganic sulphur compounds in its respiratory pathways. In fact, the details of these pathways have not yet been fully elucidated, which is indicative of the many outstanding and important questions about the biology of this bacterium that remain to be studied.

The ways in which pathogens interact with their hosts is often intricately regulated so that the bacteria can sense the right time to express genes required for colonization or virulence. One well-studied example of this is the human pathogen *Yersinia pseudotuberculosis *, which uses a plasmid-encoded Type III secretion system (T3SS) called Ysc-Yop to directly contact and deliver proteins into the cytosol of host cells during infection [10]. A plasmid-encoded master regulator, LcrF, controls directly the expression of these virulence genes, the expression of which is itself controlled by other, chromosomally encoded transcription factors, including CpxR and RscB [11]. Both of these systems are involved in sensing extracytoplasmic stresses of various kinds, through still not entirely understood mechanisms, and are found in a range of other Gram-negative pathogens, including *Escherichia coli* [12, 13]. In this issue, the group of Shiyun Chen, at the Key Laboratory of Special Pathogens and Biosafety, Wuhan, China, working with their collaborator Matthew Francis at Umeå University, Sweden, have discovered a novel interplay of these two regulatory systems [14]. Their data suggest that phosphorylated CpxR can repress promoters upstream of *rcsB*, which they demonstrate using overexpressed CpxR and also a more native physiological system in which they activate Cpx signalling through expression of a mislocalized outer membrane protein NlpE [15]. However, they are unable to detect direct binding of CpxR to these *rcs* promoters using either gel shifts or DNA footprinting, suggesting that the regulation is indirect and mediated through some other factors. However, they still present evidence for a regulatory link between the Cpx and Rsc pathways that has not been observed previously. Looking directly at the *lcrF* promoter, their data now suggest that RscB is the more important transcriptional regulator, acting as an activator, while CpxR has a milder repressing role that could be explained through its indirect effects on *rcsB*. The authors conclude by discussing why CpxR might regulate this T3SS. The Cpx response is known to downregulate the synthesis of large membrane protein complexes, like a T3SS, reducing the load on potentially damaged membranes [12], and recent evidence suggests a link between Cpx and peptidoglycan homeostasis [15, 16], which the authors suggest is another connection to the T3SS as it needs to penetrate the the peptidoglycan during its assembly.

The final paper for inclusion in the Musings this month is in the topic area of AMR and asks a simple question. If a bacterium takes up some DNA via natural transformation and that DNA contains a transposon, can this then lead to movement of the transposon onto the host chromosome? This is a relevant question as some of the most important antibiotic-resistant pathogens are naturally transformable and it is well known that antibiotic resistance genes can be carried on mobile genetic elements. Julie Kloos (@KloosJulia) with Pål Johnson (@Paal_Johnsen) from the group of Klaus Harms at the University of Norway, Tronsø, Norway, attempt to answer this question by studying Tn*1* transposition movement into *Acinetobacter baylyi* [17]. By adding this transposon to a plasmid that cannot replicate in *A. baylyi,* they detect transfer of the transposon onto the host chromosome after the plasmid was taken up by natural transformation, which is not observed when the same plasmid is electroporated into the cell or when the genes required for transposition are inactivated on the transposon. This integration after natural transformation is at low frequencies compared to other mechanisms for horizontal gene transfer, but they argue that it is biologically relevant. Interestingly, the Tn*1* inserts a non-random site on the chromosome, with a propensity for inserting around the replication terminus; the mechanistic basis for this is not yet understood, although something similar has been reported in the Tn*917* transposon when integrating into the *Bacillus subtilis* genome [18]. During natural transformation the DNA enters the cytoplasm in a single-stranded form; the authors then considered how their Tn*1* is getting onto the chromosome, and it appears that various proteins are required for plasmid recircularization. The authors also identify a role for the RecBCD system in this process, as its removal leads to significant enhancement of Tn*1* insertion. Finally, they present a model for this interesting new process, which will hopefully lead to further study, and given that transposons often carry genes for AMR any additional insight into their mechanisms of spread in bacterial populations is important.

I seem destined to not write about papers coming out of Freya Harrisons’ (@friendlymicrobe) lab at the University of Warwick, UK, as her new paper in this month’s issue has been selected as the Editor’s Choice for January. This remarkable achievement follows a previous paper from the same first author Esther Sweeney (@SweeneyEsther) which was published just last month [19]. In her new paper Esther, working with clinicians Matthew Hurley (@MatthewNHurley) and Alan Smyth (@AlanRSmyth) at the University of Nottingham, along with Marwa Hassan (@DrMarwa_Hassan) and Niamh Harrington (@_NiamhEllen) with other colleagues in Nottingham, have made further progress using a porcine lung model they developed previously [20], to now demonstrate that this also functions as a suitable model for studying how *Staphylococcus aureus* grows during chronic infections of the cystic fibrosis lung [21]. Read about the article in the *Microbiology Society* Blog, stay safe and see you next month.

Gavin Thomas

Department of Biology, University of York, York YO10 5YW, UK.
